# Hierarchically Porous, Laser-Pyrolyzed Carbon Electrode from Black Photoresist for On-Chip Microsupercapacitors

**DOI:** 10.3390/nano11112828

**Published:** 2021-10-25

**Authors:** Soongeun Kwon, Hak-Jong Choi, Hyung Cheoul Shim, Yeoheung Yoon, Junhyoung Ahn, Hyungjun Lim, Geehong Kim, Kee-Bong Choi, JaeJong Lee

**Affiliations:** 1 Nano-Convergence Mechanical Systems Research Division, Korea Institute of Machinery and Materials, 156, Gajeongbuk-Ro, Yuseong-Gu, Daejeon 34103, Korea; hakjong_choi@kimm.re.kr (H.-J.C.); scafos@kimm.re.kr (H.C.S.); ajh@kimm.re.kr (J.A.); hjlim@kimm.re.kr (H.L.); geehong@kimm.re.kr (G.K.); kbchoi@kimm.re.kr (K.-B.C.); jjlee@kimm.re.kr (J.L.); 2 Department of Nanomechatronics, Korea University of Science and Technology (UST), 217, Gajeongbuk-Ro, Yuseong-Gu, Daejeon 34113, Korea; 3 Korea Electric Power Research Institute, 105, Munji-Ro, Yuseong-Gu, Daejeon 34056, Korea; yyoon@kepco.co.kr

**Keywords:** laser-pyrolyzed carbon, direct laser writing, microsupercapacitor, photoresist

## Abstract

We report a laser-pyrolyzed carbon (LPC) electrode prepared from a black photoresist for an on-chip microsupercapacitor (MSC). An interdigitated LPC electrode was fabricated by direct laser writing using a high-power carbon dioxide (CO_2_) laser to simultaneously carbonize and pattern a spin-coated black SU-8 film. Due to the high absorption of carbon blacks in black SU-8, the laser-irradiated SU-8 surface was directly exfoliated and carbonized by a fast photo-thermal reaction. Facile laser pyrolysis of black SU-8 provides a hierarchically macroporous, graphitic carbon structure with fewer defects (I_D_/I_G_ = 0.19). The experimental conditions of CO_2_ direct laser writing were optimized to fabricate high-quality LPCs for MSC electrodes with low sheet resistance and good porosity. A typical MSC based on an LPC electrode showed a large areal capacitance of 1.26 mF cm^−2^ at a scan rate of 5 mV/s, outperforming most MSCs based on thermally pyrolyzed carbon. In addition, the results revealed that the high-resolution electrode pattern in the same footprint as that of the LPC-MSCs significantly affected the rate performance of the MSCs. Consequently, the proposed laser pyrolysis technique using black SU-8 provided simple and facile fabrication of porous, graphitic carbon electrodes for high-performance on-chip MSCs without high-temperature thermal pyrolysis.

## 1. Introduction

On-chip microsupercapacitors (MSCs) have received great interest as miniaturized power sources due to their compact size, high power, and long life cycle characteristics as supercapacitors (SCs) with micron-scale electrode dimensions [1,2,3]. As MSCs employ an in-plane interdigitated electrode (IDE) for efficient ion transport across the neighboring electrodes, they are considered promising thin-film energy storage devices for wearable, stretchable, and textile electronics [4,5,6].

Recently, carbon nanomaterials (such as carbon nanotubes [7], graphene [8], and MXene [9]) have attracted tremendous interest as high-performance on-chip MSCs electrode materials due to their large surface area and high electrical conductivity. However, these materials require a costly and complicated material preparation process, such as high-temperature synthesis, material purification, and dispersion in toxic solvents for further processing [7,8,9].

On the other hand, pyrolyzed carbons (PCs) derived from photoresists have advantages over carbon nanomaterials in terms of process compatibility with semiconductor processes (e.g., spin coating, photolithography, direct laser writing, etc.) [10,11,12]. As conventional photoresists show an electrically insulating property, a thermal annealing process is required to carbonize and convert photoresists to electrically conductive carbon. The conventional carbonization process, called thermal pyrolysis, adopts thermal furnace heating at high temperatures in a controlled gas environment. However, the thermal pyrolysis process requires costly equipment and long process times. Furthermore, thermally pyrolyzed carbon (TPC) shows moderate electrical conductivity due to low crystallinity with an amorphous structure and low surface area due to volumetric shrinkage during thermal pyrolysis [13,14,15]. Despite the precise pattern of a lithographically defined IDE, the TPC showed limited electrochemical performance as an MSC electrode.

As an alternative carbonization process, laser pyrolysis has recently been recognized as a facile pyrolysis strategy due to ultrafast photo-thermal reaction by high-power laser irradiation [16,17,18,19]. Laser pyrolysis has, however, rarely been employed for the carbonization of photoresists due to the low absorption of photon energy and the conversion efficiency of photons to thermal energy in conventional ultraviolet (UV) resins. A specific photoresist was carbonized by a high-power infrared (IR) laser with a long wavelength [16]. Recently, the J. Tour group reported high-resolution laser-induced graphene (LIG) from a commercial photoresist [18]. However, the application of LPC derived from photoresists has still not been reported.

In this work, laser-pyrolyzed carbon (LPC) from a commercial photoresist is proposed as a high-performance on-chip MSC electrode. A composite of SU-8 and carbon black, called black SU-8, was used as a novel precursor of an LPC electrode. Laser irradiation of a spin-coated black SU-8 by a high-power CO_2_ laser generated a three-dimensional, hierarchically porous carbon electrode with an expansion ratio of 3.3. Optimized laser irradiation conditions resulted in high-crystallinity graphitic carbon with fewer defects (I_D_/I_G_ = 0.19). The resultant on-chip MSC electrode based on an LPC with a finger number of 16 showed a large areal specific capacitance of 1.26 mF cm^−2^ at a scan rate of 5 mV s^−1^ as well as an excellent stability of 94.7% retention of the initial capacitance after 4000 cycles.

## 2. Experimental Setup

### 2.1. Materials

A black SU-8 photoresist (GMC 1040, Gersteltec Sarl, Pully, Switzerland), an SU-8-based negative tone photo-epoxy material, is composed of SU-8 and carbon black materials. Polyvinyl alcohol (PVA) and phosphoric acid (H_3_PO_4_) were purchased from Sigma Aldrich (Seoul, Korea).

### 2.2. Material Preparation

First, a silicon dioxide (SiO_2_) layer (2 μm thickness) was deposited on a 4-inch silicon (Si) wafer by wet oxidation as an insulating layer. A black SU-8 was spin-coated with 2000 rpm to fabricate a 3 μm thick, black photoresist layer on the SiO_2_/Si wafer. After the spin-coating process, a further baking and exposure process followed the black SU-8 processing protocol provided by the manufacturer.

For the laser pyrolysis process, the black SU-8 film was irradiated with a continuous CO_2_ laser (wavelength: 10.6 μm) using a laser engraving system (Model: C30, Coryart, Seoul, Korea) in an air environment. Direct laser writing of the high-power CO_2_ laser was conducted to simultaneously carbonize and define an IDE pattern, resulting in an LPC-IDE. The scanning power (*P*) and speed (*v*) were optimized to investigate the effect of these parameters on the material and device performance. The gap between the scan steps was set to be 0.025 mm. To improve the wettability of LPC with an electrolyte, the LPC-IDE was treated with an oxygen plasma process. The oxygen plasma treatments (150 W, 5 min) were performed by a plasma system (Cograde, Femto Science Co., Hwaseong-si, Korea).

As-fabricated LPC-IDEs on various substrates were electrically connected to the electrochemical measurement system via copper (Cu) conductive tape. As a gel electrolyte, 1 M PVA in H_3_PO_4_ aqueous electrolyte was prepared and drop casted onto an LPC-IDE.

### 2.3. Characterization

A scanning electron microscope (SEM, Hitachi, Tokyo, Japan) analysis was performed to investigate the structural characterization. Raman spectroscopy (Renishaw, Wotton-under-Edge, UK) was conducted to analyze the crystallinity and defect level of the LPC. An X-ray photoelectron spectroscopy (XPS, Thermo Fisher Scientific, Waltham, MA, USA) analysis was carried out to evaluate the chemical bonding structure. A four-point probe system was used to measure the sheet resistance at room temperature.

All electrochemical measurements were performed with a two-electrode configuration. Cyclic voltammetry (CV), galvanostatic charge-discharge (CD), and electrochemical impedance spectroscopy (EIS) measurements were carried out using a potentiostat (Model: VSP, BioLogic Inc., Seyssinet-Pariset, France). The CV measurement was conducted at an operating voltage (0–0.8 V) with various scan rate ranges (5–1000 mV s^−1^). The CD measurement was carried out with current densities ranging from 2 μA cm^−2^ to ~100 μA cm^−2^. The EIS testing was performed using the sinusoidal signal of 5 mV amplitude at a frequency range of 10 mHz–1 MHz. The areal specific capacitance (*C_A_*) from CV measurement was based on the following Equation [20]:

(1) $$C_{A}=\frac{1}{2\times A\times \nu \times \left(V_{f}-V_{i}\right)}\int_{V_{i}}^{V_{f}}I\left(V\right)dV$$

where *A* is the total electrode area (cm^2^) of the LPC-IDE in the MSC, *ν* is the scan rate (V s^−1^), *V_f_* and *V_i_* are the voltage limits of the CV testing, and *I(V)* is the measured current (A) at different voltages.

The areal specific capacitance (*C_A_*) from CD measurement was calculated by the following Equation [20]:

(2) $$C_{A}=\frac{I}{A\times dV/dt}$$

where *I* is the discharge current (A), *A* is the total electrode area (cm^2^) of the LPC-IDE in the MSC, and *dV/dt* is the discharge slope (V s^−1^) of the CD curves.

## 3. Results and Discussions

**Figure 1:**
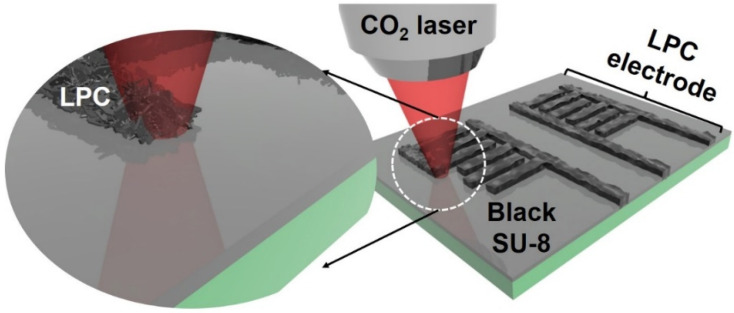
A schematic diagram demonstrating the process used to fabricate an LPC electrode based on a black SU-8 materials spin-coated on a SiO_2_/Si wafer.

Figure 1 illustrates the fabrication process of hierarchically porous LPC electrodes for on-chip MSCs. A high-power CO_2_ laser (λ: 10.6 μm) was used to directly carbonize various carbon precursor materials such as polymers and metal organic frameworks (MOFs) [21,22,23]. Most photoresists have low absorption of a long-wavelength laser, preventing the photoresist from being carbonized by simple laser irradiation [24,25]. However, because the black SU-8 film showed improved absorbance against the long-wavelength laser, the CO_2_ laser irradiation on a spin-coated black SU-8 film caused a photo-thermal reaction (Figure S1). The carbon black materials in black SU-8 acted as optical absorbers. A black SU-8 spin-coated on a SiO_2_/Si wafer showed a flat and highly stacked film with a thickness of 3 μm (Figure S2).

**Figure 2:**
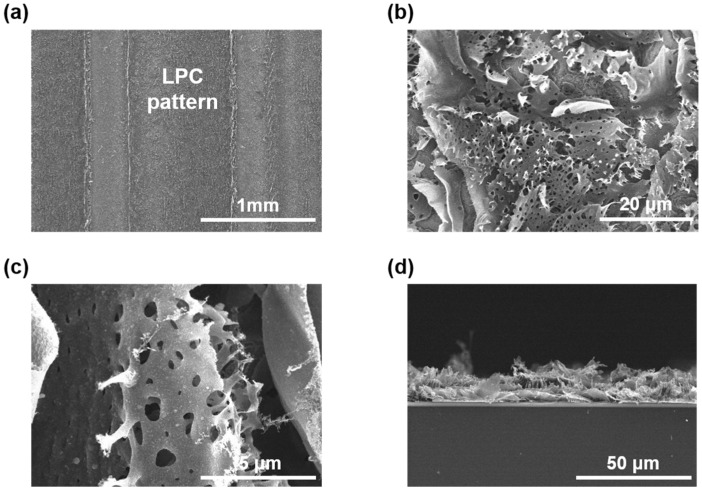
SEM images of LPC-IDE. (**a**–**c**) Top-view images of LPC-IDE. LPC showed a hierarchically porous structure with macro- and meso-pores. (**d**) Cross-sectional images of LPC-IDE. A highly exfoliated LPC structure was observed after laser pyrolysis.

Figure 2 shows SEM images of an LPC film after CO_2_ laser irradiation. The black SU-8 surface irradiated by a CO_2_ laser showed a rough morphology with a porous structure. The highly stacked black SU-8 film (*t*: 3 μm) was expanded to be a porous LPC film of 10 μm in thickness. The expansion ratio, defined by the thickness ratio between the expanded LPC and black SU-8 film, was found to be 3.3. During the photo-thermal reaction, oxygen functional groups on the surface of black SU-8 tend to convert to gases such as H_2_O, CO, and CO_2_. The gaseous by-products escape from the highly stacked black SU-8 film during laser irradiation, resulting in a porous LPC film with volume expansion. The pore diameter observed in an LPC film ranged from 0.2 to 3 μm, indicating that most pores were micro or meso-pores favorable to facile ion transport for supercapacitors (Figure 2b,c). The large volume expansion caused by laser pyrolysis is noticeably different from the volume shrinkage by conventional thermal pyrolysis. During thermal pyrolysis, the loss of photoresist materials generally occurs due to gradual evaporation of gaseous by-products upon decomposition of PR. While the relatively slow heating speed in thermal pyrolysis guarantees escape of the gaseous product by diffusion, a fast heating speed in laser pyrolysis generates large expansion in the highly stacked film prior to diffusion [26,27]. This volume expansion of black SU-8 after laser pyrolysis is similar to that of graphene oxide (GO) and polyimide (PI) with high absorbance of a CO_2_ laser [28,29].

**Figure 3:**
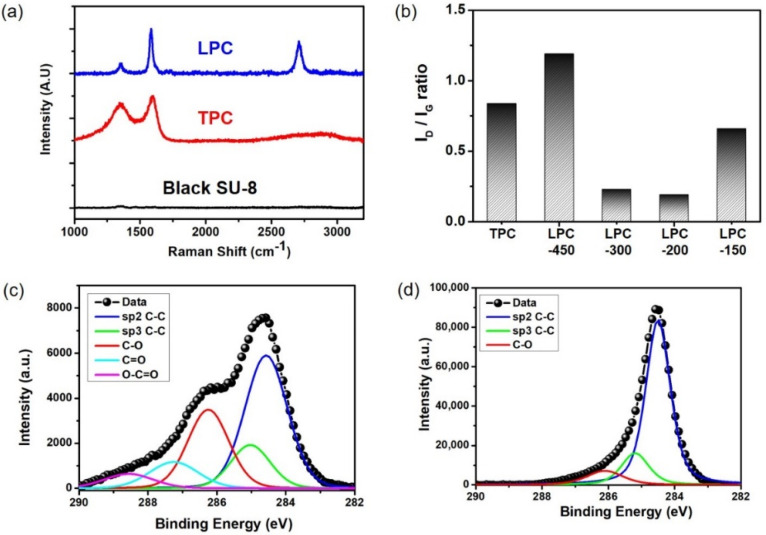
(**a**) Raman spectra of a typical black SU-8, TPC, and LPC film. (**b**) I_D_/I_G_ ratio of TPC and LPC-x samples to compare the crystallinity of the conductive carbons derived from the black SU-8 resins. The LPC sample was denoted as LPC-x, where x stands for scanning speed of CO_2_ laser pyrolysis. C1s XPS spectra of (**c**) black SU-8 and (**d**) LPC films.

Raman spectroscopy is a representative measurement used to investigate the defects and crystallinity of carbon materials [30]. Figure 3a shows the Raman spectra of LPC, TPC, and black SU-8. A TPC sample was fabricated by the high-temperature (1000 °C) thermal annealing process in a tube furnace with an inert argon gas (flow rate: 500 sccm). While the Raman spectrum of black SU-8 without laser irradiation shows broad photoluminescence without a noticeable peak in the G (~1580 cm^−1^) and D bands (~1350 cm^−1^), the LPC showed three distinct peaks in the G, D, and 2D bands (~2700 cm^−1^). The I_D_/I_G_ ratio of black SU-8 after laser irradiation decreased notably, indicating that high-power CO_2_ laser pyrolysis altered the defects and crystal structure of black SU-8. The spectrum of the TPC also showed a graphitic D-peak and G-peak with an I_D_/I_G_ ratio of 0.85. The I_D_/I_G_ ratio of TPC was consistent with those (~1.0) of other amorphous TPCs with large defects [31,32]. The I_D_/I_G_ ratio of LPC was calculated to be 0.23, which is similar to that of reduced GO and other laser-reduced graphene [33,34,35]. The 2D peak is typically used to differentiate graphitic carbons from amorphous carbons [36]. The appearance of the 2D peak in the LPC spectrum showed that high-power CO_2_ laser irradiation produced graphitic carbon from the photoresist rather than amorphous carbon. Generally, carbon precursors such as GO and PI irradiated by a high-power CO_2_ laser could reach extremely high localized temperatures (~2500 °C) by lattice vibration [37]. The high localized temperature of the black SU-8 could thus induce graphitization of the LPC, similar to the effect of a high-temperature thermal annealing process.

We also investigated the effect of the laser irradiation conditions on the graphitic structures of the LPC films (Figure S3). In the Raman spectra of LPC films prepared by different laser scanning speeds, the I_D_/I_G_ ratio was decreased to 0.19 as the scanning speed was decreased to 200 mm/s. At the lowest speed of 150 mm/s, the I_D_/I_G_ ratio was increased to 0.66 due to partial oxidation by the accumulated thermal energy in the air (Figure 3b). The I_2D_/I_G_ ratio was relatively consistent under the applied scanning speed (Table S1). The sharp 2D pattern with a small full width at half maximum of 50–60 cm^−1^ and I_2D_/I_G_ ratio (~0.7) indicated that the LPC showed 2D graphite structures with randomly stacked multiple graphene layers. Thus, CO_2_ laser irradiation on the black SU-8 resulted in a graphitized LPC film with a small I_D_/I_G_ ratio and an obvious 2D peak contrary to the TPC film by conventional thermal pyrolysis.

XPS measurements were performed to characterize the chemical bonding states of black SU-8 and LPC films (Figure 3c,d). The C1 spectra of black SU-8 showed large amounts of oxygen functional groups from the epoxy-based SU-8 material as well as sp2 C–C (284.6 eV) and sp3 C–C bonds (285.5 eV) [38]. Meanwhile, the C 1s spectra of the LPC after laser pyrolysis presented three distinct carbon bond peaks: sp2 C–C, sp3 C–C, and C–O (286.5 eV). These results demonstrated that the oxygen functional groups in the black SU-8 material were significantly removed and that large amounts of sp2 C–C and sp3 C–C were formed by laser pyrolysis. Laser irradiation conditions affected the sheet resistance of the LPC films. At a relatively low scanning speed (16 W, 450 mm/s), the sheet resistance increased to 500 Ω sq^−1^ with a large standard deviation (20%). As the scanning speed decreases, the average and standard deviation of the LPC decrease to 105 Ω sq^−1^ and 7%, respectively. Moreover, an increase in laser power improved the sheet resistance of the LPC films (Figure S4). The sheet resistance of the LPC under optimal laser irradiation conditions was comparable (~100 Ω sq^−1^) to that of previously reported TPC films [39,40,41].

**Figure 4:**
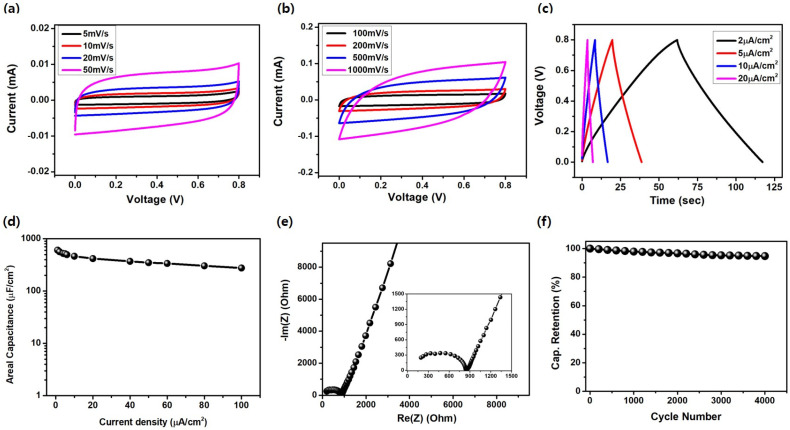
(**a**,**b**) CV curves of the LPC-MSC at various scan rates. (**c**) CD curve of the LPC-MSC at various current densities. (**d**) Areal specific capacitance calculated from CD graphs as a function of current density. (**e**) Nyquist plot and (**f**) cyclic test result of the LPC-MSC under CD testing at a large current density of 20.0 μA cm^−2^.

Figure 4 shows the results of electrochemical characterization of a typical LPC-MSC. A typical electrode width (W_e_), the gap between electrodes (W_g_), the length (L), and the number of finger electrodes (*N*) of an LPC-IDE were chosen as 0.9 mm, 0.3 mm, 2 mm, and 16, respectively. The scanning power, speed, and gap between the scan steps were 16 W (Watt), 300 mm/sec, and 0.025 mm. Quasi-rectangular shapes in CV curves were observed in the scan rates ranging from 5 mV s^−1^ to 1000 mV s^−1^, indicating the LPC-MSC has a typical electric double-layer capacitor (EDLC) characteristic. The areal specific capacitance (*C_A_*) calculated from the CV graph was measured to be 1.26 mF cm^−2^ and 0.38 mF cm^−2^ at scan rates of 5 mV s^−1^ and 1000 mV s^−1^, respectively (Figure 4a,b). From the galvanostatic CD testing, the LPC-MSCs also exhibited electrochemically capacitive behavior with nearly triangular CD curves in a wide range of current densities (2–100 μA cm^−2^) (Figure 4c,d). The *C_A_* from the CD testing was calculated to be 0.56 mF cm^−2^ and 0.30 mF cm^−2^ at current densities of 2 μA cm^−2^ and 100 μA cm^−2^, respectively. The *C_A_* (0.30–0.56 mF cm^−2^) of the LPC-MSC is comparable to those (0.05–0.50 mF cm^−2^) of previous MSCs based on photoresist-derived carbon reported for on-chip energy storage applications [12,13,41,42].

Electrochemical impedance spectroscopy (EIS) testing was carried out to analyze the ion transport behavior of the LPC-MSC. A nearly vertical slope was shown in the low frequency region of the EIS graph, confirming double-layer characteristics. At the high-frequency region, small Warburg and semi-circular regions were observed by indicating low charge-transfer resistance and efficient ion transport owing to the porous LPC structure (Figure 4e, inset). The equivalent series resistance (ESR), defined by the resistance obtained from the intersection of the straight line of the EIS graph, was measured to be 850 Ω, comparable to those of the TPC electrodes [12,13,41]. The electrochemical stability of the LPC-MSC was tested under CD testing at a large current density of 20 μA cm^−2^ (Figure 4f). The stability test result showed around 94.7% capacitance retention after 4000 cycles, confirming the good cyclic stability.

**Figure 5:**
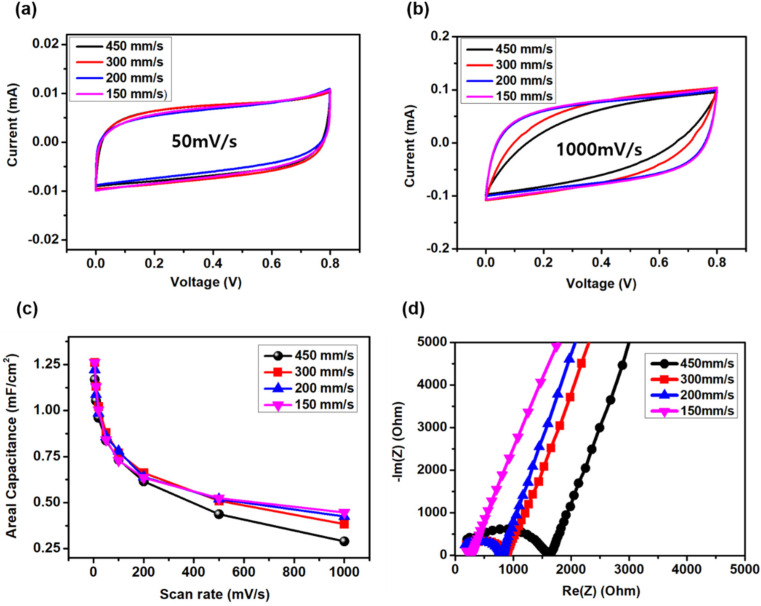
Electrochemical performances of LPC-MSCs (N = 16) with different scanning speeds (450, 300, 200, and 150 mm/s). (**a**,**b**) CV curves at the scan rates of (**a**) 50 mVs^−1^ and (**b**) 1000 mV^−1^. (**c**) Areal specific capacitance calculated from CV graphs as a function of scan rate. (**d**) Nyquist plots of the LPC-MSCs.

As the scanning speed significantly affected the electrical conductivity of the resulting LPC films, we investigated the effect of the scanning speed on the electrochemical performance of the LPC-MSCs. Four LPC-MSCs were fabricated with different scanning speed (450, 300, 200, and 150 mm/s) conditions. The CV graphs of the four MSCs showed nearly rectangular shapes with the same integral area at a low scan rate (5 mV/s), indicating that all MSCs had good double-layer capacitor characteristics with the same electrode capacitance (Figure 5a). At a high scan rate (1000 mV/s), the LPC-MSC with the lowest scanning speed still maintained a rectangular shape EDLC (Figure 5b). Meanwhile, the LPC-MSCs with faster scanning speed showed distorted CV graphs at a high scan rate, resulting in degradation of the capacitance. The distortion in CV graph of LPC-450 means that LPC-450 had a large ESR, including electrode resistance and ion transport resistance. In terms of the time constant of capacitor charging, the LPC-450 shows the largest time constant among LPC-x samples. Noting that the LPC-450 showed the largest electrode resistance in Figure S4, the electrode resistance affected the time constant of capacitor charging and the capacitance at fast scan rate of CV testing.

The rate capabilities of the four MSCs were similar; however, the MSC with the lower scanning speed showed a better rate performance (Figure 5c). The Nyquist plots from EIS testing depicted similar vertical slopes for all MSCs, demonstrating that the LPC film showed facile ion transport due to the macroporous electrode structure. In particular, the lower scanning speed showed a lower ESR at the high-frequency region (Figure 5d). This low ESR characteristic resulted from the low sheet resistance of the graphitic LPC film with fewer defects.

**Figure 6:**
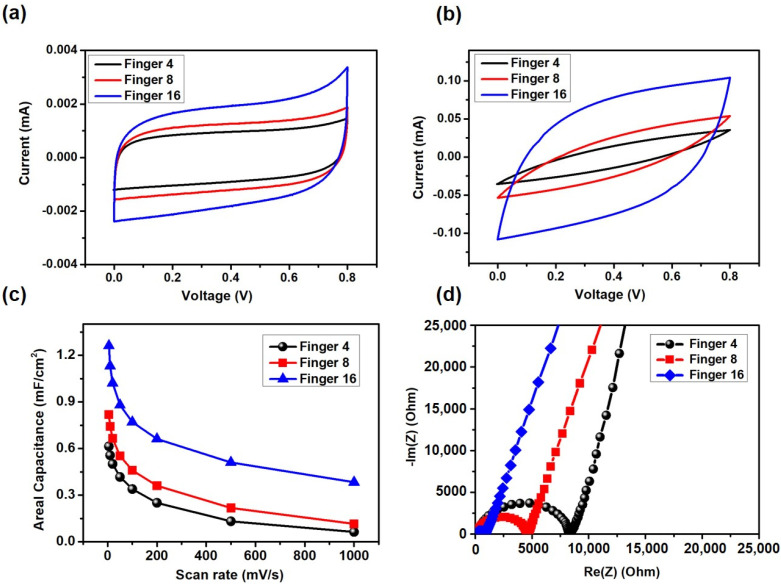
Electrochemical performances of LPC-MSCs (W_e_ /W_g_ = 1) with different finger numbers (N: 4, 8, and 16). (**a**,**b**) CV curves at the scan rates of (**a**) 50 mVs^−1^ and (**b**) 1000 mV^−1^. (**c**) Areal specific capacitance calculated from CV graphs as a function of scan rate. (**d**) Nyquist plots of the LPC-MSCs.

We also compared the electrochemical performances of LPC-MSCs with different finger numbers (*N*). These LPC-MSCs with the same device area and W_e_/W_g_ ratio (~1) resulted in finger numbers (*N*) of 4, 8, and 16. The laser irradiation condition to make LPC-MSCs was set to be 16 W (Watt) and 300 mm/s in the scanning power and speed. The MSC with a larger *N* and smaller W_e_ maintained the ideal rectangular shape at scan rates ranging from 50 to 1000 mV s^−1^. On the other hand, the MSC with a smaller *N* and larger W_e_ showed a distorted CV graph at the high scan rate of 1000 mV s^−1^ (Figure 6a,b). The MSC with a larger *N* exhibited the best rate capability among the three MSCs. These results indicate that a small W_e_ by high-resolution patterning was critical for facile ion transport at fast charging operations (Figure 6c). In the Nyquist plot, it was observed that the semi-circular region was significantly reduced as the finger number increased. As the semi-circular region indicates low resistance of the charge transfer and efficient transport of the electrolyte ion in the electrode, the MSC with a larger *N* shows a better rate capability while maintaining capacitance at fast scan rates.

## 4. Conclusions

We demonstrated the high performance of MSCs based on an LPC electrode derived from black SU-8. An LPC film was fabricated by CO_2_ laser pyrolysis of a spin-coated black SU-8 film. Due to the high absorption of carbon blacks in black SU-8, the black SU-8 film was simultaneously carbonized and patterned to fabricate an LPC-IDE by direct laser writing. Facile laser pyrolysis of black SU-8 provides a hierarchically porous carbon structure with a graphitic structure and a low degree of defects, contrary to conventional amorphous carbon from photoresist by thermal pyrolysis. The experimental conditions of CO_2_ direct laser writing were optimized to fabricate high-quality LPCs for supercapacitor electrodes with fewer defects and good porosity. The typical MSC based on LPC-IDEs showed large areal capacitance compared with most MSCs from TPC by thermal pyrolysis of conventional photoresists. In addition, the results revealed that the high-resolution electrode pattern in the same device area as that of LPC-MSCs significantly affected the rate performance of the MSCs. Consequently, the proposed laser pyrolysis strategy using black SU-8 provided simple and facile fabrication of porous, graphitic carbon electrodes for high-performance on-chip MSCs without high-temperature thermal pyrolysis.

## Data Availability

The data presented in this study are available from the corresponding author upon request.

## Supplementary Materials

The following are available online at https://www.mdpi.com/article/10.3390/nano11112828/s1, Figure S1: An FT-IR analysis of a bare SU-8 and a black SU-8 film. From the FT-IR curve, the black SU-8 film showed improved absorbance against the wavelength (10.6 μm) of the CO_2_ laser. Figure S2: (a) Top view and (b) cross-sectional view of a SEM image of a black SU-8 film spin-coated on a SiO_2_/Si wafer. SEM images showed a flat and highly stacked SU-8 film (*t*: 3 μm). Pictures of (c) a black SU-8 film and (d) an LPC pattern after CO_2_ laser pyrolysis on 4″ SiO_2_/Si wafer. Figure S3: Raman spectra of LPC films prepared by different laser scanning speeds. A constant laser power (*P*: 16 W) was applied. Table S1: Analysis of I_D_/I_G_ and I_2D_/I_G_ of LPC films with different laser irradiation conditions. Figure S4: A histogram showing the sheet resistance of LPCs according to the scanning speed during CO_2_ laser pyrolysis process.
