# Supplementary material for: Hierarchically Porous, Laser-Pyrolyzed Carbon Electrode from Black Photoresist for On-Chip Microsupercapacitors
Source: Nanomaterials (Basel). 2021 Oct 25;11(11):2828. doi: 10.3390/nano11112828 (PMC8620280; doi:10.3390/nano11112828)
Supplement: Supplementary file 1 [file nanomaterials-11-02828-s001.zip › nanomaterials-1404451-supplementary.pdf]

nanomaterials

Supplementary Materials

Hierarchically Porous, Laser-Pyrolyzed Carbon Electrode from Black Photoresist for On-Chip Microsupercapacitors

Soongeun Kwon [1,*], Hak-Jong Choi [1], Hyung Cheoul Shim [1,2], Yeoheung Yoon [3], Junhyoung Ahn [1], Hyungjun Lim [1,2], Geehong Kim [1], Kee-Bong Choi [1] and JaeJong Lee [1,2]

[1] Nano-Convergence Mechanical Systems Research Division, Korea Institute of Machinery and Materials, 156, Gajeongbuk-Ro, Yuseong-Gu, Daejeon 34103, Korea; hakjong_choi@kimm.re.kr (H.-J.C.); scafos@kimm.re.kr (H.C.S.); ajh@kimm.re.kr (J.A.); hjlim@kimm.re.kr (H.L.); geehong@kimm.re.kr (G.K.); kbchoi@kimm.re.kr (K.-B.C.); jjlee@kimm.re.kr (J.L.)

[2] Department of Nanomechatronics, Korea University of Science and Technology (UST), 217, Gajeongbuk-Ro, Yuseong-Gu, Daejeon 34113, Korea;

[3] Korea Electric Power Research Institute, 105, Munji-Ro, Yuseong-Gu, Daejeon 34056, Korea; yyoon@kepco.co.kr

* Correspondence: sgkwon@kimm.re.kr

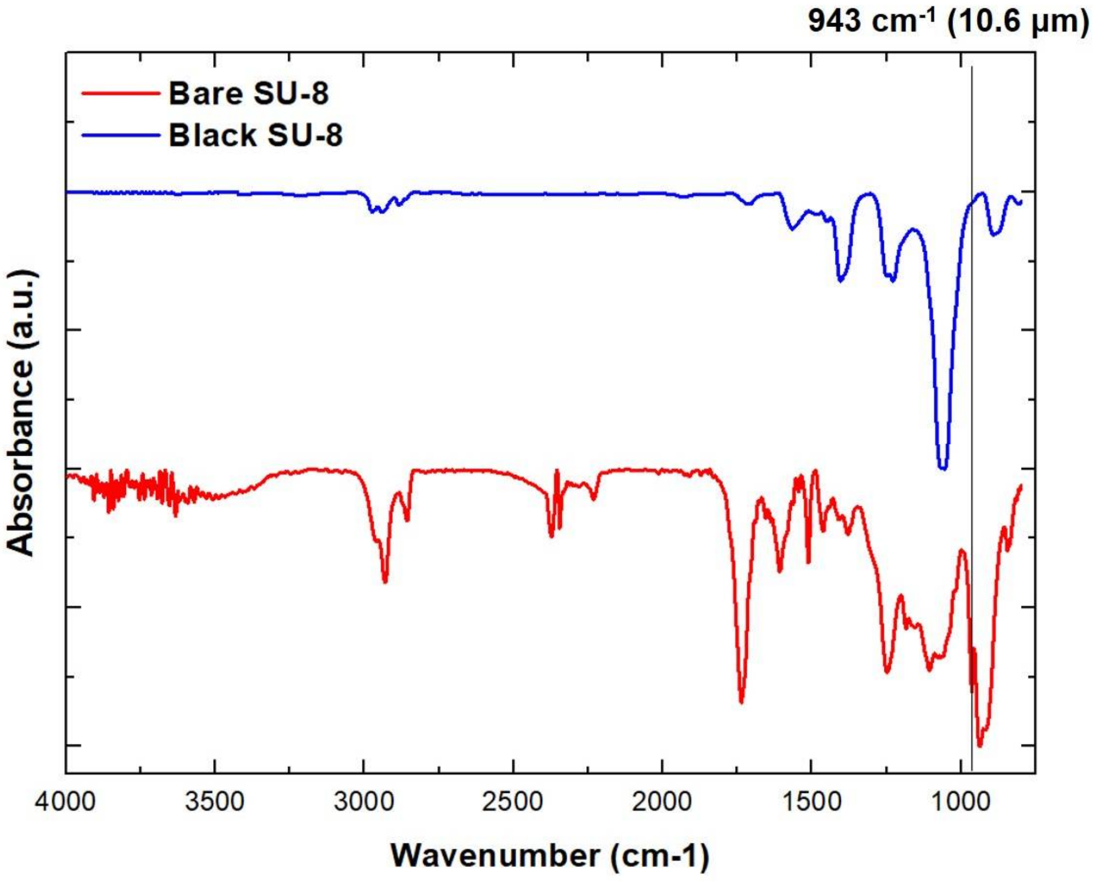

Figure S1. A FT-IR analysis of a bare SU-8 and a black SU-8 films. From the FT-IR curve, the black SU-8 film showed improved absorbance against the wavelength (10.6 μm) of CO_2 laser.

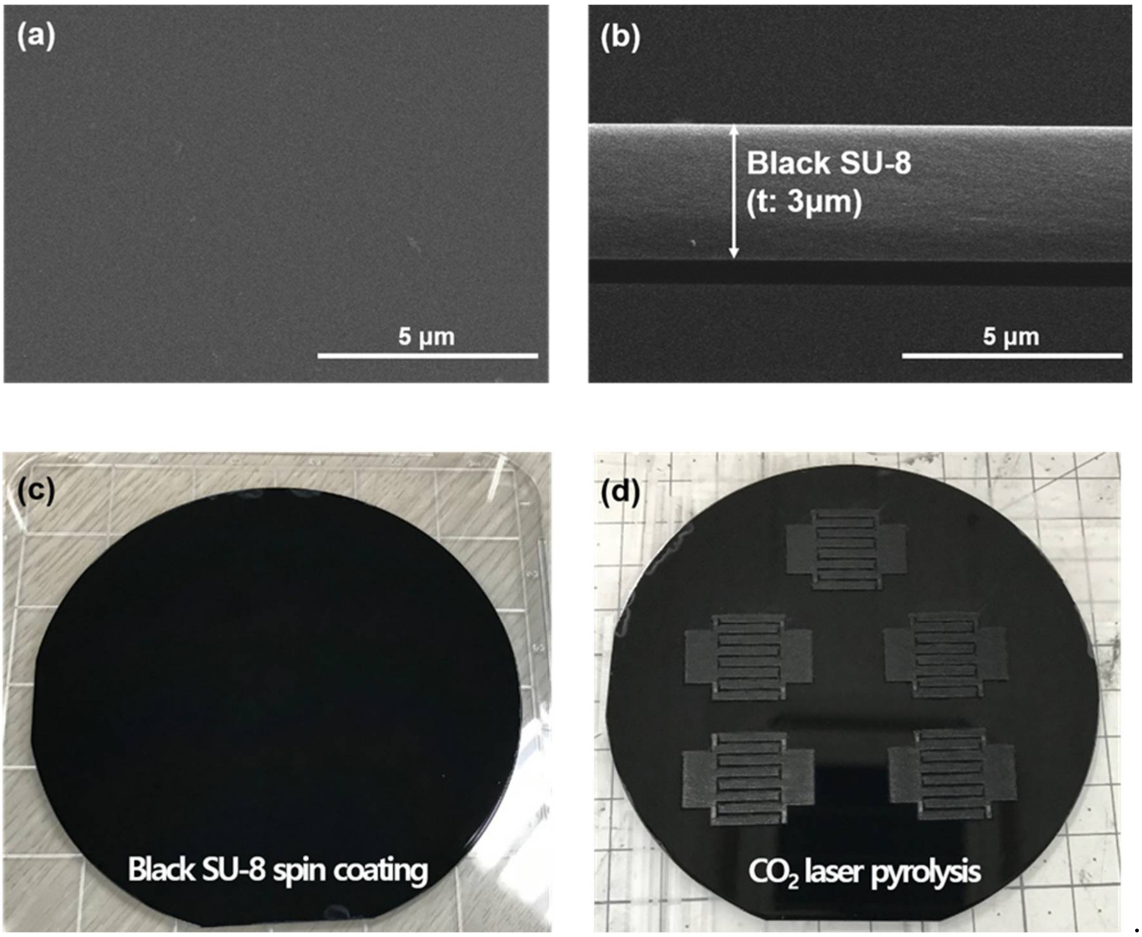

Figure 2. (**a**) Top view and (**b**) cross-sectional view of a SEM image of a black SU-8 film spin-coated on a SiO_2/Si wafer. SEM images showed a flat and highly packed SU-8 film (*t*: 3μm). Pictures of (**c**) a black SU-8 film and (**d**) a LPC pattern after CO_2 laser pyrolysis on 4'' SiO_2/Si wafer.

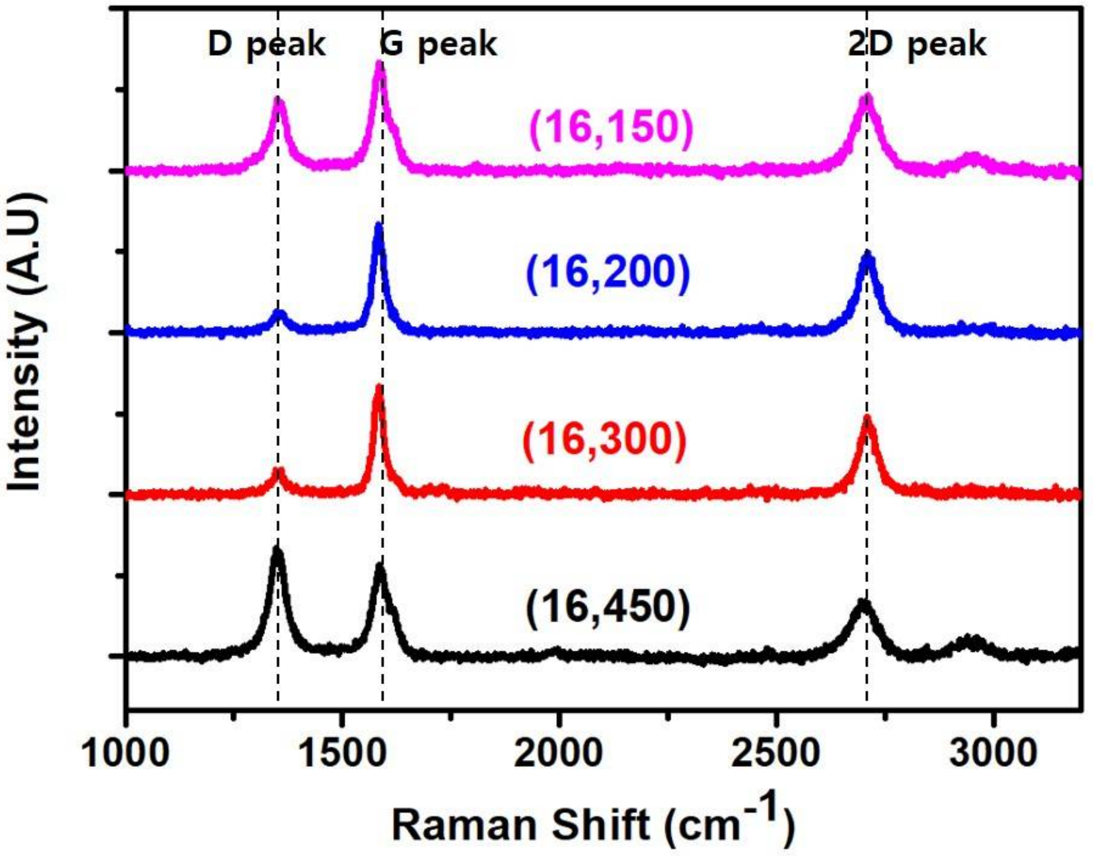

Figure S3. Raman spectra of LPC films prepared by different laser scanning speed. A constant laser power (*P*: 16W) was applied.

Table S1. Analysis of I_D/I_G and I_{2D}/I_G of LPC films with different laser irradiation conditions.

Samples (Power (W), Speed (mm/sec))	I_D/I_G	I_{2D}/I_G
(16, 450)	1.19	0.61
(16, 300)	0.23	0.72
(16, 200)	0.19	0.74
(16, 150)	0.66	0.70

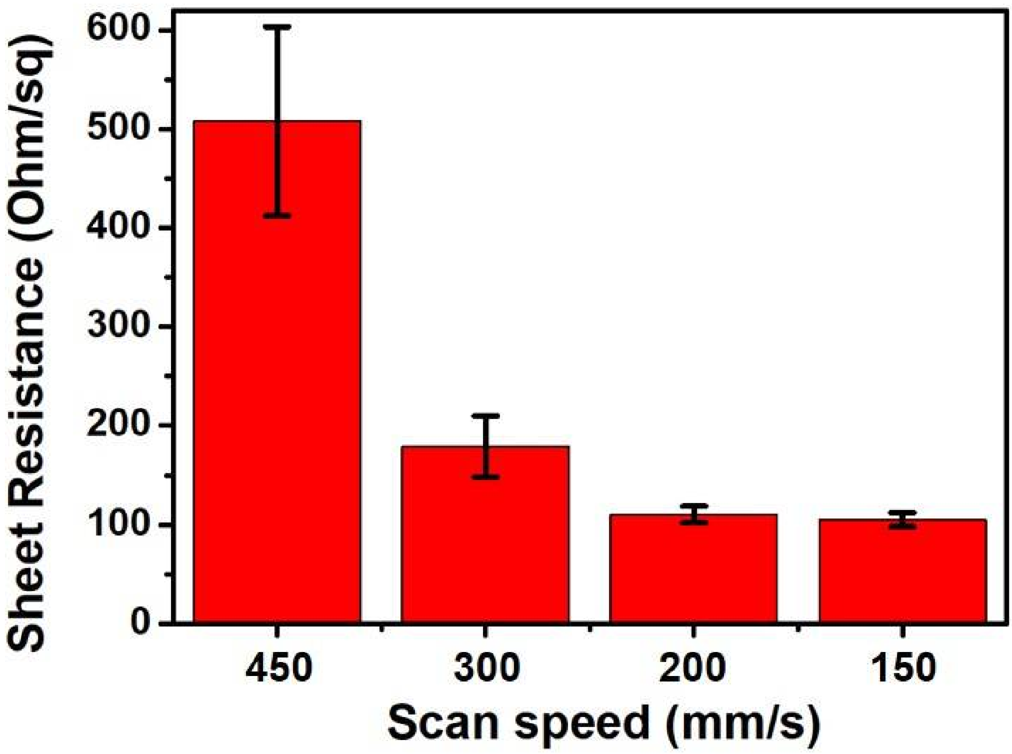

Figure S4. A histogram showing the sheet resistance of LPCs according to the scanning speed during CO_2 laser pyrolysis process.
